# Characteristics and origin of the relatively high-quality tight reservoir in the Silurian Xiaoheba Formation in the southeastern Sichuan Basin

**DOI:** 10.1371/journal.pone.0180980

**Published:** 2017-07-07

**Authors:** Xiaoxing Gong, Zejin Shi, Yong Wang, Yaming Tian, Wenjie Li, Lei Liu

**Affiliations:** 1 College of Energy Resources, Chengdu University of Technology, Chengdu, China; 2 State Key Laboratory of Oil and Gas Reservoir Geology and Exploitation, Chengdu University of Technology, Chengdu, China; 3 College of Earth Sciences, Chengdu University of Technology, Chengdu, China; East China Normal University, CHINA

## Abstract

A mature understanding of the sandstone gas reservoir in the Xiaoheba Formation in the southeastern Sichuan Basin remains lacking. To assess the reservoir characteristics and the origin of the high-quality reservoir in the Xiaoheba Formation, this paper uses systematic field investigations, physical property analysis, thin section identification, scanning electron microscopy and electron microprobe methods. The results indicate that the Xiaoheba sandstone is an ultra-tight and ultra-low permeability reservoir, with an average porosity of 2.97% and an average permeability of 0.56×10^−3^ μm^2^. This promising reservoir is mainly distributed in the Lengshuixi and Shuangliuba regions and the latter has a relatively high-quality reservoir with an average porosity of 5.28% and average permeability of 0.53×10^−3^ μm^2^. The reservoir space comprises secondary intergranular dissolved pores, moldic pores and fractures. Microfacies, feldspar dissolution and fracture connectivity control the quality of this reservoir. The relatively weak compaction and cementation in the interbedded delta front distal bar and interdistributary bay microfacies indirectly protected the primary intergranular pores and enhanced late-stage dissolution. Late-stage potassium feldspar dissolution was controlled by the early-stage organic acid dissolution intensity and the distance from the hydrocarbon generation center. Early-stage fractures acted as pathways for organic acid migration and were therefore important factors in the formation of the reservoir. Based on these observations, the area to the west of the Shuangliuba and Lengshuixi regions has potential for gas exploration.

## 1. Introduction

Tight sandstone gas is a prominent component of oil and gas resources. By 2014, tight sandstone gas accounted for 17% of the U.S. total gas production and 31.6% of China’s total gas production [1]. The boundary of a tight sandstone gas reservoir is a relative concept, for which the partitioning standard varies by country, resource conditions and technical conditions at different points in time [2–4]. Currently, a tight sandstone gas play with a porosity of less than 4% and a permeability less than 0.1% is worth exploration and development. Typical examples of tight sandstone gas plays include Jurassic strata with porosities of 0.09% to 4.9% and permeabilities of 0.014×10^−3^ to 8.35×10^−3^ μm^2^ in the East Texas Basin [5–6], Carbonaceous strata with porosities of 2.2% to 6.8% and permeabilities of 0.013×10^−3^ to 2.04×10^−3^ μm^2^ in the Alpine Foreland Basin [6–7], Upper Devonian-Mississippian strata with porosities of 0.7% to 5.6% and permeabilities of 0.103×10^−3^ to 2.14×10^−3^ μm^2^ in the Appalachian basin [6, 8], and Cretaceous strata with porosities of 1.2% to 5.8% and permeabilities of 0.056×10^−3^ to 0.96×10^−3^ μm^2^ in the San Juan Basin [6, 9]. In the Ordos Basin of China, the lower limits of the physical properties of a tight reservoir are a porosity of 2% to 4% and a permeability of 0.01×10^−3^ μm^2^ to 0.1×10^−3^ μm^2^ [10]. To determine whether the deeply buried tight sandstone of the Xujiahe Formation (Xu-2 Member) in the western Sichuan Basin has exploration potential, a reservoir porosity of more than 4% must be measured [11]. Therefore, the presence of a relatively high-quality reservoir with a porosity of greater than 4% and a permeability greaterthan 0.01×10^−3^ μm^2^ is of particular importance to natural gas exploration in tight clastic rocks in the Sichuan Basin.

In the southeastern Sichuan Basin, Silurian marine clastic rocks are moderately buried and feature a good source-reservoir-cap rock assemblage, making them a good exploration target [12–14]. The Xiaoheba Formation is an important reservoir in Silurian strata. Previously published studies on the Xiaoheba Formation have focused on favorable reservoir plays and the depositional filling process [15–20], sequence stratigraphy [21, 22], and material source [23, 24]. Research on the Xiaoheba Formation reservoir has focused on reservoir parameter evaluation [25], diagenesis [26], and discussion of the diagenetic evolution and densification [27, 28] of the tight sandstone. The majority of the research has not focused on the relatively high-quality reservoirs. The general tightness of the Xiaoheba sandstone represents a high-risk target for gas exploration. Furthermore, few studies have evaluated the existence of relatively high-quality reservoir enrichment zones and thefactors controlling their development in this area.

Based on previous studies, this paper places emphasis on typical outcrops of the Silurian Xiaoheba Formation at the southeastern margin of the Sichuan Basin, and discusses the characteristics of tight sandstone reservoirs and influencing factors of relatively high-quality reservoirs of the Xiaoheba Formation through the analysis of physical properties, thin section, cathodoluminescence images scanning electron microscope (SEM) observations, and electron microprobe data. Moreover, the developmental mode of relatively high-quality reservoirs is proposed to enable expansion of natural gas exploration in the southeastern Sichuan Basin.

## 2. Geologic setting

**Fig 1:**
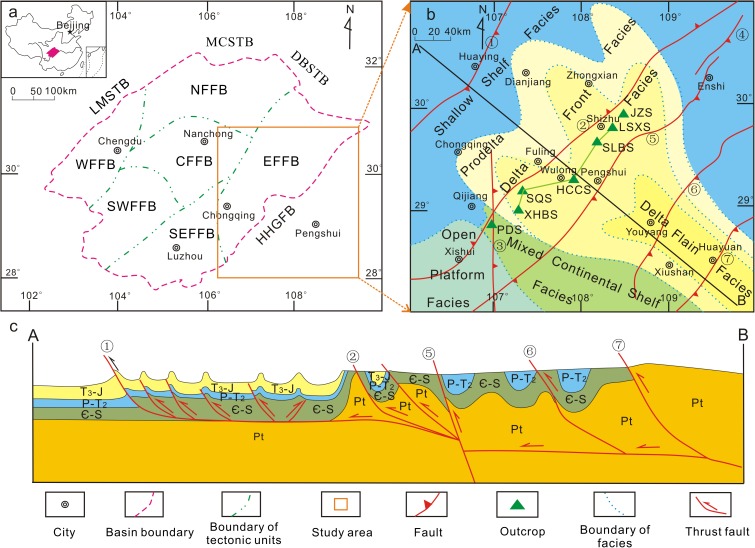
Geological maps of the southeastern Sichuan Basin. a. Structural location of the study area (Modified from Liu et al., 2014) [29]. b. Location of field outcrops of the Silurian Xiaoheba Formation along the periphery of the southeastern Sichuan Basin (Basemap modified from Jin, 2010) [30]. c. Synthetic structural section (line ‘A–B’ in Fig 1b) from Huaying to Huayuan (Modified from Yan et al., 2003) [31]. Numbers from ① to ⑦ represent the following faults: Huayingshan, Qiyueshan-Xishui, Zunyi-Pingba, Jianshi, Pengshui, Hefeng-Longshan, and Huayuan, respectively. MCS: Micangshan Tectonic Belt, DBSTB: Dabashan Tectonic Belt, LMSTB: Longmenshan Tectonic Belt, WFFB: Western fault-fold belt in the Sichuan Basin, NFFB: Northern fault-fold belt in the Sichuan Basin, SWFFB: Southwestern fault-fold belt in the Sichuan Basin, SEFFB: Southeastern fault-fold belt in the Sichuan Basin, EFFB: Eastern fault-fold belt in the Sichuan Basin, CFFB: Central fault-fold belt in the Sichuan Basin, HHGFB: Huannan-Hubei-Guizhou Fold Belt, PDS: Podu section, XHBS: Xiaoheba section, SQS: Sanquan section, HCCS: Huangcaochang section, SLBS: Shuangliuba section, LSXS: Lengshuixi section, JZS: Jinzhu section, T3-J: Upper Triassic to Jurassic, P-T_2_: Permian to Middle Triassic, Є-S: Cambrian-Silurian, Pt: Proterozoic.

**Fig 2:**
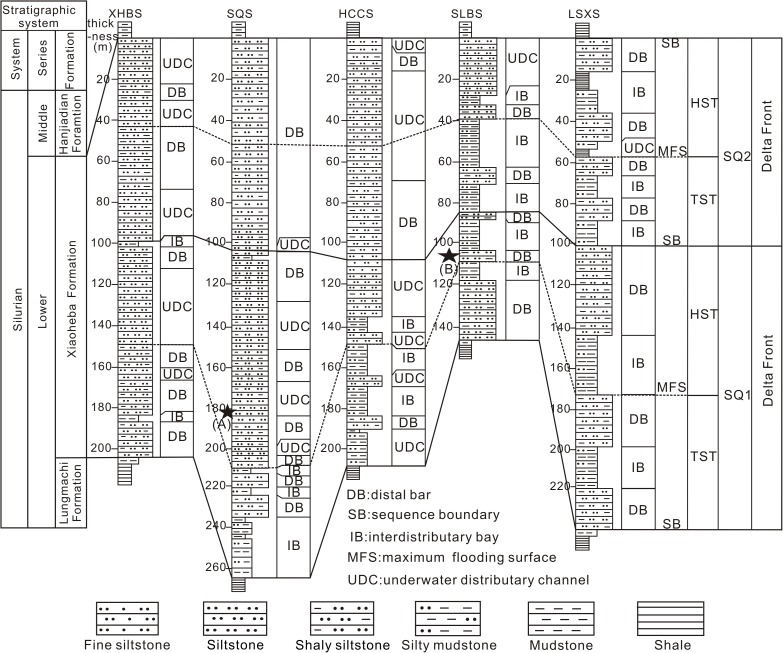
Correlation of survey sections of the Silurian Xiaoheba Formation in the southeastern Sichuan Basin (See Fig 1B for the location of each section).

The southeastern Sichuan Basin is situated to the west of the Qiyueshan-Xishui fault and east of the Huayingshan fault. The structures in this region for a high angle belt fold and thrust in the eastern Sichuan Basin and a low angle fold and thrust belt in the southern Sichuan Basin (Fig 1A and 1B). Lower Paleozoic strata crop out to the west of the Huayuan fault and east of the Qiyueshan-Xishui fault (Fig 1C). The Caledonian Guangxi tectonic activity uplifted and eroded the Silurian strata, resulting in the absence of upper Silurian strata. Consequently, the lower Silurian Longmaxi and Xiaoheba formations and the middle Silurian Hanjiadian Formation were successively deposited. The black shale present in the lower member of the Longmaxi Formation is a high-quality source rock, and the Xiaobahe Formation is interpreted to have been deposited within a transitional delta depositional system (Fig 1C and Fig 2). The lithology is composed of silty fine-grained sandstone and argillaceous siltstone interbedded with silty mudstone and shale, which act as the primary reservoir strata. The thick stratified middle Silurian Hanjiadian shale interbedded with thinly laminated siltstone acts as a good cap rock. The availability of good source-reservoir-cap rock conditions makes the Xiaoheba sandstone an important target layer for Silurian gas exploration in the southeastern Sichuan Basin.

## 3. Methodology

All the samples were obtained from five survey sections (Xiaoheba, Sanquan, Huangcaochang, Shuangliuba, and Lengshuixi section) and two observed sections (Podu and Jinzhu). The sections feature the delta front subfacies (Fig 2), although the Podu section also features mixed platform facies.

Analyses of the lithofacies and diagenesis are based on 202 thin section samples, including 48 samples stained with blue epoxy resin under vacuum (prepared by the State Key Reservoir Geology and Development Engineering Lab of the Chengdu University of Technology), 24 samples stained with red epoxy resin under vacuum (prepared by the Huayang Testing Center of the Sichuan Geology and Mineral Bureau), and 130 ordinary samples with double-sided polishing and no cover slip. The samples were carefully observed using a Nikon ECLIPSE LV100POL polarizing microscope at the Geosciences Institute of the Chengdu University of Technology. Some were stained with a mixture of alizarin red and potassium ferricyanide while being observed under a polarizing microscope. A total of 30 samples were selected for carbon film coating under vacuum. Microscopic rock features were observed using a JSM-5500LV SEM at the Petroleum Geology Testing Center of the Research Institute of Exploration and Development, Zhongyuan Oilfield Company, SINOPEC, using a 20 kV acceleration voltage and a 0.6 A beam current.

To determine the rock reservoir physical properties, plugs that were 2.5 cm in diameter and 2.5 cm in length were prepared from 189 fresh sandstone samples for porosity and permeability measurements using a GDS-9F gas permeability tester in the State Key Reservoir Geology and Development Engineering Lab of the Chengdu University of Technology. Ten typical samples were selected to evaluate the pore structures using the AutoPore IV 9505 mercury injection apparatus at the Petroleum Geology Testing Center of the Research Institute of Exploration and Development, Zhongyuan Oilfield Company, SINOPEC.

To determine feldspar dissolution types and cement features, 20 thin section samples were selected for observation of the mineral and cement types using the CL8200MK5 cathodoluminescence apparatus at the Energy Institute of the Chengdu University of Technology, using a 12 kV acceleration voltage and a 300 μA beam current. Furthermore, 48 feldspar particles were measured using a Nikon ECLIPSE LV100POL polarizing microscopy and coated with carbon film under vacuum. These samples were then analyzed with an EPMA-1720 electron microprobe apparatus equipped with a backscattered electron detector (BSE) using a 20 kV acceleration voltage and a 10–15 μA beam current at the Geosciences Institute of the Chengdu University of Technology.

## 4. Results

### 4.1. Petrological features

**Fig 3:**
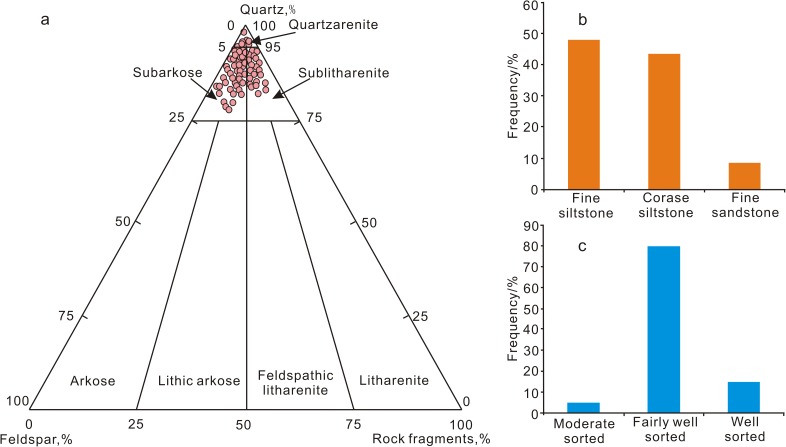
Rock composition, lithology and sorting distribution of the Xiaoheba Formation sandstone. a. Classification of the sandstone samples using Folk's classification [32], b. lithology distribution and c. sorting distribution.

According to the test results of the 202 thin sections, feldspathic quartzarenite and lithic quartzarenite, followed by quartzarenite are the dominant lithologies (Fig 3A). The quartz content in the clastic components ranges from 77% to 98%, with an average of 87.77% and is dominated by single-crystals with partial secondary quartz enlargement. The feldspar content ranges from 2% to 16%, with an average of 7.73%, and is dominated by plagioclase, followed by microcline and orthoclase, with feldspar dissolution and partial calcite replacement has occurred. The lithic content ranges from 2% to 14%, with an average of 5.77%, and is dominated by mica group fragments and occasional phyllite fragments. The cements are dominated by carbonate and silica. Granularity analysis indicates the dominance of siltstone and fine sandstone (Fig 3B). The sandstone has a moderate to relatively high component maturity, is fairly well sorted (Fig 3C), has generally moderate to good roundness, and a relatively low textural maturity.

### 4.2. Physical properties

**Fig 4:**
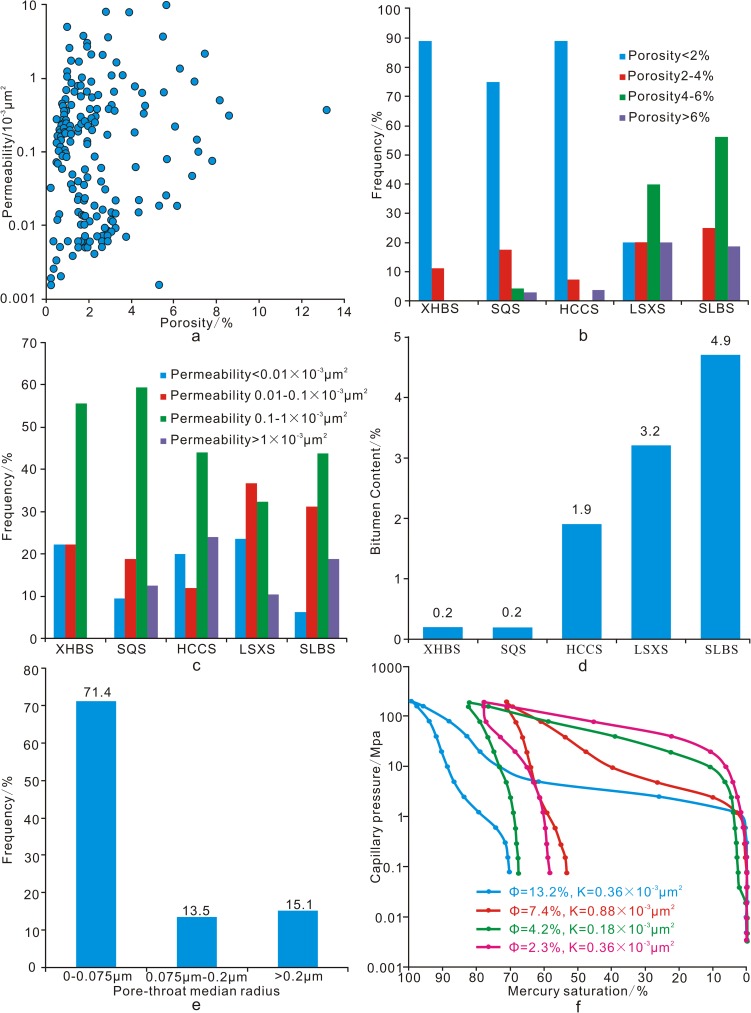
Characteristics of the Xiaoheba Formation sandstone properties. a. Crossplot of porosity and permeability for the Xiaoheba Formation. b. Isogram of porosity frequency of the outcrops. c. Histogram of permeability frequency for the outcrops. d. Histogram of average bitumen content for the outcrop thin sections. e. Distribution of the pore throat radius, Shuangliuba section. f. Typical mercury injection curve feature, Shuangliuba section.

According to the 189 analyses of the Xiaoheba sandstone, the porosity ranges from 0.04% to 13.23%, with an average of 2.97%, and the permeability ranges from 0.0006×10^−3^ to 7.9724×10^−3^ μm^2^ with an average of 0.56×10^−3^ μm^2^. Most of the samples have a porosity of less than 2% and a permeability of less than 1×10^−3^ μm^2^, reflecting the ultra-tight and ultra-low permeability nature of this tight sandstone reservoir (porosity < 5% and 0.1×10^−3^ μm^2^ < permeability< 1×10^−3^ μm^2^). The porosity and permeability are poorly matched (Fig 4A).

Based on previous studies of the Xujiahe sandstone reservoir (Xu-2 Member) in the western Sichuan Basin [11] and characteristics of the Xiaoheba sandstone in this study, we defined the lower limits of the porosity and permeability of this reservoir as 2% and 0.1×10^−3^ μm^2^, respectively, and defined a relatively high-quality reservoir as one having a porosity greater than 4%. Based on this scale, the reservoirs are mainly developed in the Lengshuixi and Shuangliuba regions, and relatively high-quality reservoirs are distributed primarily in the Shuangliuba region, which features reservoirs with an average porosity of 5.28% and an average permeability of 0.53×10^−3^ μm^2^ (Fig 4B and 4C). The Lengshuixi and Shuangliuba sections exhibit significantly higher average bitumen contents than the other sections (Fig 4D).

Mercury injection analysis of samples from the Shuangliuba region indicates that the displacement pressure ranges from 2.52 to 19.18 MPa, the maximum pore-throat radius ranges from 0.06 to 0.5 μm, the median pressure ranges from 4.07 to 106.97 MPa (Fig 4E) and the median pore-throat radius is 0.01–0.20 μm. Approximately 71.35% of the pore throats have a median radius less than 0.075 μm, 15.13% have a median radius greater than 0.20 μm, and 13.52% have a median radius between 0.075 and 0.2 μm (Fig 4F). The pore-throat system in this reservoir is dominated by micro pore-throats and fine pore-throats. The mercury curves indicate that the pore-throat system in the sandstone is complex and that the pores associated with relatively higher porosity show good sorting and high skewness (skewness describes the weighting of the pore-throat radius distribution towards a larger or smaller size relative to the mean value) (Fig 4F). These observations reveal relatively good storage and migration conditions for hydrocarbons.

### 4.3. Pore space

**Fig 5:**
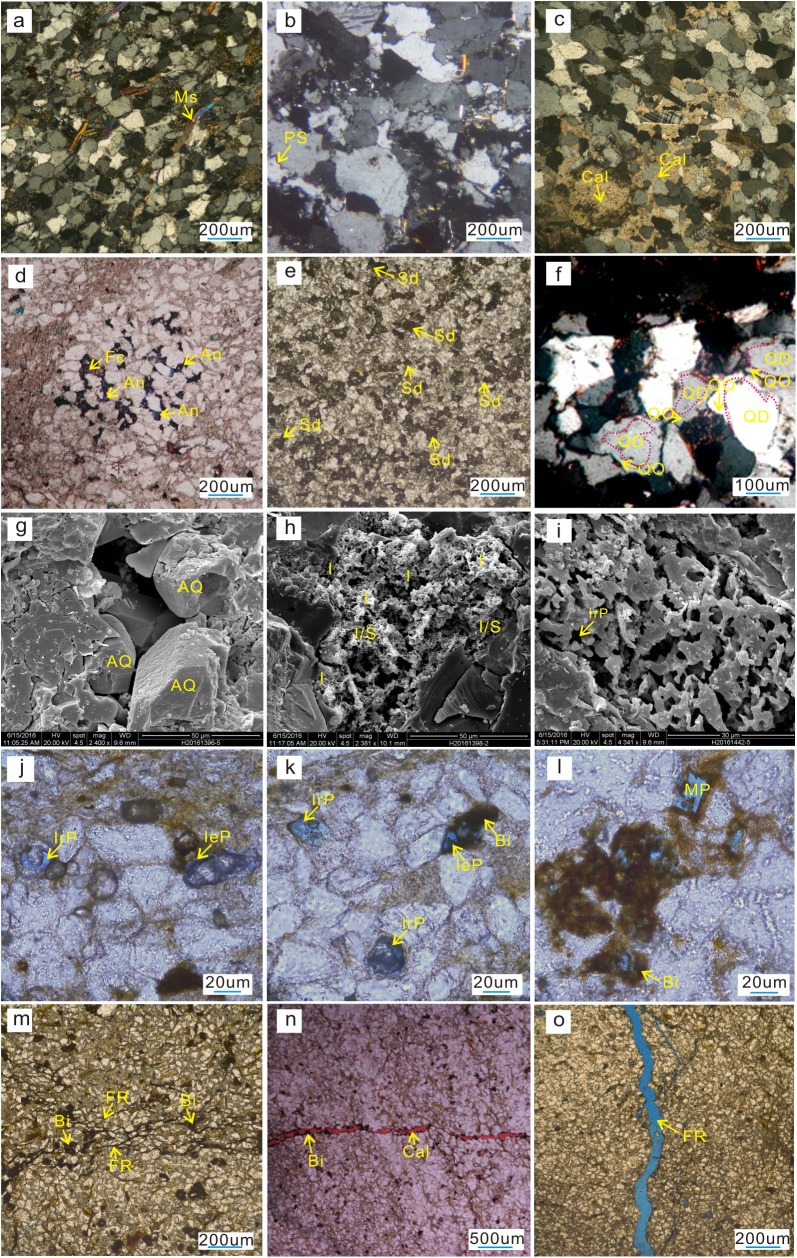
Microscopic texture and reservoir space features of the Silurian Xiaoheba Formation reservoir in the southeastern Sichuan Basin. a. Micrograph of thin section showing muscovite sheets that are directionally arranged and bent (arrow), damaged particles in mosaic contact, Jinzhu section, Shizhu. b. Micrograph of a thin section showing a stylolitic contact between particles and intense pressure dissolution effects (arrow), Huangcaochang Section, Wulong. c. Micrograph of a thin section showing calcite cementation in a fine sandstone (arrow), Huangcaochang section, Wulong. d. Micrograph of a thin section showing intergranular pores and dissolved pores filled with ferrocalcite (red) and ankerite (blue), Xiaoheba section, Nanchuan, stained with alizarin red and potassium ferricyanide. e. Micrograph of a thin section showing authigenic siderite cementation in a feldspathic quartzarenite (arrow), Jinzhu section, Shizhu. f. Micrograph of a thin section showing quartz overgrowths (arrow), Lengshuixi section, Shizhu. g. SEM micrograph showing intergranular pore space filled with authigenic quartz, Podu section, Qijiang. h. SEM micrograph showing mixed-layer illite/smectite and illite between clastic particles, Huangcaochang section, Wulong. i. SEM micrograph showing skeleton-shaped feldspar particles formed by dissolution, with well-developed dissolved micro-pores (arrow), Shuangliuba section, Shizhu. j. Micrograph of a thin section showing intragranular pores and intergranular pores, Shuangliuba section, Shizhu, blue stained thin section. k. Micrograph of a thin section showing intragranular dissolved pores and intergranular dissolved pores filled with bitumen, Shuangliuba section, Shizhu, blue stained thin section. l. Micrograph of a thin section showing intragranular dissolved pores and moldic pores associated with feldspar, Shuangliuba section, Shizhu. m. Micrograph of a thin section showing fracture completely filled with bitumen, Shuangliuba section, Shizhu. n. Micrograph of a thin section showing a fracture filled with calcite and some bitumen, Xiaoheba section, Nanchuan, stained with alizarin red and potassium ferricyanide. o. Micrograph of a thin section showing an unfilled fracture, Shuangliuba section, Shizhu, blue stained thin section. Ms-muscovite; PS-pressure dissolution; Cal-Calcite; Fc-ferrocalcite; An-ankerite; Sd-siderite; QD-detrital quartz; QO-quartz overgrowths; QA-authigenic quartz; I-illite; I/S-mixed-layer illite/smectite; IrP-intragranular pores; IeP-intergranular pores; MP-moldic pores; Bi-bitumen; FR-fracture.

The thin section examination and the cathodoluminescence and SEM observation indicate strong compaction (Fig 5A) and intense pressure dissolution (Fig 5B) in the Xiaoheba sandstone. Additionally, the particles have been cemented with calcite (Fig 5C), ferrocalcite (Fig 5D), ankerite (Fig 5D), siderite (Fig 5E), and quartz overgrowths, which are easy to discriminate from the detrital grains due to the existence of some dust rims (Fig 5F). Furthermore, the pore space has been filled with authigenic quartz and clay minerals (Fig 5G and 5H), resulting in a tight lithology and an absence of primary pores. However, some reservoir space remains l available in an ordinary tight context, such as intragranular dissolved pores (Fig 5I, 5J and 5k), intergranular dissolved pores (Fig 5J and 5K), moldic pores (Fig 5L) and fractures (Fig 5M, 5N and 5O). The dissolved particles are mainly feldspar, and fractures are relatively well developed in the Lengshuixi and Shuangliuba regions.

## 5. Discussion

### 5.1. Microfacies

**Fig 6:**
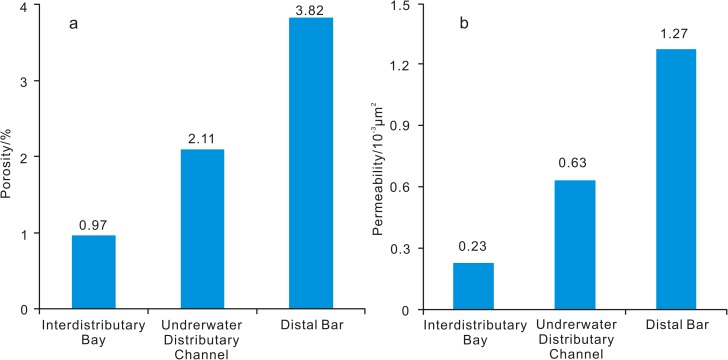
Histogram showing the distribution of physical properties among different sedimentary microfacies in the Silurian Xiaoheba Formation, in the southeastern Sichuan Basin.

The surveyed sections correspond to the delta front subfacies (Fig 1C and Fig 2) and contain deposits associated with interdistributary bay, underwater distributary channel, and developed distal bar microfacies. Due to relatively weak hydrodynamic conditions, sandstone is fine grained and rich in plastic particles (mica group) and clay minerals (averaging 15%). The ordinary porosity and permeability of sandstone has been damaged to varying degrees due to the effects of compaction, resulting in an increase in the tightness of the reservoir. Statistical data from the Xiaoheba Formation (Fig 6A and 6B) indicate that the distal bar microfacies mainly consists of siltstone or argillaceous siltstone and is vertically interbedded with the interdistributary bay microfacies. The interdistributary bay mudstone and silty mudstone have preserved the porosity. As a result, the porosity averages 3.82%, the permeability averages 1.27×10^−3^ μm^2^. The underwater distributary channel is composed mainly of siltstone and very thick sand bodies, which were modified by destructive diagenetic processes, such as compaction and cementation, during burial. Therefore, in this microfacies, the porosity averages 2.11%, the permeability averages 0.63×10^−3^ μm^2^. The interdistributary bay microfacies is usually composed of silty mudstone and mudstone and contains very fine-grained sediments. Thus, the porosity averages less than 1% and the permeability averages 0.23×10^−3^ μm^2^.

**Fig 7:**
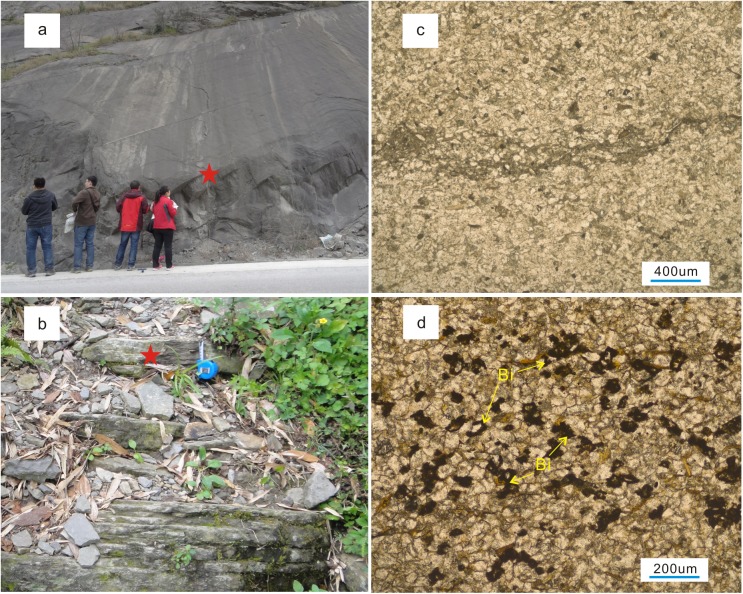
Relationship between pores and structures in field sections of the Silurian Xiaoheba Formation in the southeastern Sichuan Basin. a. Thick-bedded massive siltstone, Sanquan section, Nanchuan (corresponding to point ‘A’ in Fig 2). b. Laminated siltstone interbedded with silty mudstone, Shuangliuba section, Shizhu (corresponding to point ‘B’ in Fig 2). c. Strong compaction, tight rock (location: star in Fig 7A). d. Relatively weak compaction, dissolved pores filled with bitumen (location: star in Fig 7B).

Macroscopically, compaction and cementation are quite evident in the thick-bedded sandstone sections. However, no reservoirs are present in the thick-bedded sandstone in the Huangcaochang, and Nanchuan regions (Fig 7A and 7C). In the Shuangliuba and Lengshuixi regions, reservoirs are present in the form of sandstone units interbedded with mudstone units with approximately equal thicknesses (in places, the sandstone thickness is less than the silty mudstone or mudstone thickness) (Fig 2). This interbedded sandstone and mudstone structure protected the sand bodies from significant compaction during early diagenesis and the presence of the muddy interbeds hindered the drainage of fluids from the sandstone during compaction. As a result, primary intergranular pores have been indirectly preserved, thereby laying the foundation for later-stage dissolution (Fig 7B and 7D).

## 5.2. Feldspar dissolution

Secondary pores generated by dissolution modify the tight physical properties and formation of high-quality reservoirs. Genetic mechanisms for secondary dissolution suggested by previous researchers include organic acid dissolution [33–38], dissolution of carbonate cements by CO_2_ [39, 40], meteoric freshwater leaching [41–45], clay mineral transformation [46–48], and quartz dissolution in an alkaline environment [49–51].

**Fig 8:**
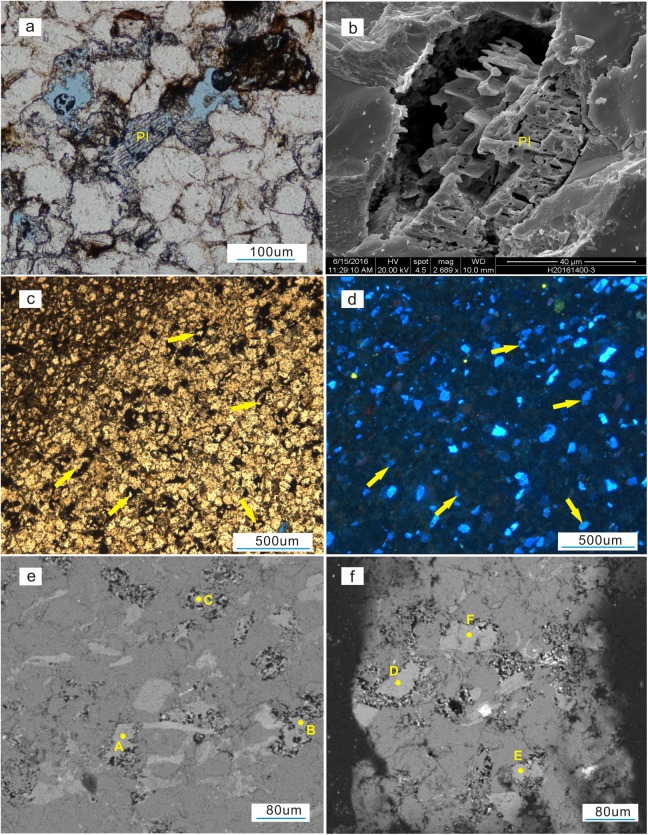
Microscopic dissolution features in Silurian Xiaoheba Formation grains from the southeastern Sichuan Basin. a. Micrograph of a thin section showing the partially and completely dissolved plagioclase, Shuangliuba Section, Shizhu. b. SEM micrograph showing partially dissolved plagioclase, Shuangliuba Section, Shizhu. c. Partially dissolved grains with bitumen filling the pore space, Shuangliuba section, Shizhu. d. Cathodoluminescence features of Fig 8C. e. BSE image showing dissolved grains and electron microprobe analysis points, Shuangliuba section, Shizhu. f. BSE image showing dissolved grains andmicroprobe analysis points, Shuangliuba section, Shizhu. Pl-Plagioclase.

**Fig 9:**
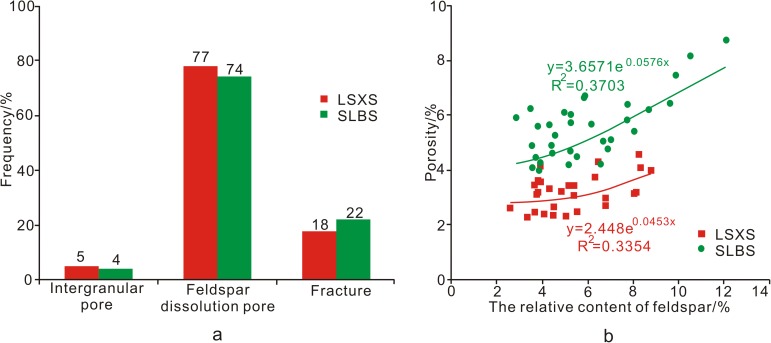
Relationship between porosity and feldspar dissolution. a. Pore type frequencies in the Shuangliuba and Lengshuixi sections; b Relationship between porosity and the relative content of feldspar. SLBS-Shuangliuba section; LSXS- Lengshuixi section.

In this study, the micrographs show that dissolution occurred along cleavage fissures in the plagioclase (Fig 8A), some of which turned into moldic pores (Fig 5L). An SEM micrograph also shows that feldspars were partially dissolved, forming skeleton-like structures (Fig 8B). The margins of the feldspar grains have been partially dissolved, and the resulting pore space has been filled with hydrocarbons. The residual feldspars appear blue under cathodoluminescence (Fig 8C and 8D). BSE images show that the grains have formed bay-like features (Fig 8E and 8F), and the electron microprobe data indicate that the dissolved grains are potassium feldspar (Table 1). The pore type frequency statistics for the Shuangliuba and Lengshuixi sections show that secondary dissolved feldspar pores account for the majority of the pore space (Fig 9A) and the porosity is positively correlated with the feldspar content (Fig 9B). Hence, feldspar dissolution is closely related to high-quality reservoirs. However, the formation of dissolved feldspar pores is complicated due to the complex burial and uplift history of the Xiaoheba Formation, and to investigating the dissolution of this feldspar is of great importance.

**Table 1:** Residual dissolved mineral components based on electron microprobe analyses (unit: ppm).

Test Point	Na_2_O	K_2_O	Cr_2_O_3_	Al_2_O_3_	CaO	MnO	MgO	SiO_2_	FeO	NiO	TiO_2_
**A**	673	14822	33	19068	20	51	0	59253	317	59	23
**B**	1013	15048	0	18692	58	0	0	60235	9	37	20
**C**	864	14993	0	18951	18	12	6	59381	125	0	18
**D**	966	14917	9	18779	16	27	0	61271	335	0	14
**E**	198	10563	25	27957	7	616	2603	42086	4251	44	941
**F**	736	15151	0	18242	0	27	0	59838	60	46	0

Previous research has indicated that potassium feldspar has a higher free energy increment (△G) of dissolution than anorthite and albite [52] and that it is difficult to dissolve in an open or semi-open system. Moreover, the Xiaoheba sandstone is a marine clastic rock, and potassium feldspar dissolution can be impaired in an open or semi-open system with marine-sourced potassium-rich pore fluids. Lithological profile observations of the Xiaoheba Formation in the field reveal the absence of unconformity surfaces and epigenetic meteoric leaching dissolution, and the thin section observations have revealed no proof of hypergene dissolution processes, despite the uplift of the Silurian strata in the southeast Sichuan Basin during the Guangxi tectonic event prior to the burial of the Xiaoheba Formation during the late Permian to Late Triassic. Therefore, the Xiaoheba Formation was not exposed, during the uplift event or modified during the period of hydrocarbon generation and migration in the underlying Longmaxi Formation source rocks [53]. Additionally, many pores are filled with illite (Fig 5H). Based on a burial depth of over 6500 m and a maximum paleo-temperature exceeding 180°C for the Xiaoheba sandstone, the mechanism of feldspar dissolution may be related to the dissolution of plagioclase by organic acids and the dissolution of potassium feldspar dissolution at high temperatures.

During the depositional period, anorthite and other alkali feldspars are dissolved during the transport process in low-temperature open systems (Reaction 1) and completely disappear during the depositional stage or the initial stage of burial diagenesis. Secondary pores are rarely preserved due to later diagenetic modification.

$$CaAl_{2}Si_{2}O_{8}\left(anorthite\right)+2H^{+}+H_{2}O=Al_{2}Si_{2}O_{5}{\left(OH\right)}_{4}\left(kaolinite\right)+Ca^{2+}$$ (1)

From the early stage of burial diagenesis (temperatures of approximately 60°C) to temperatures of 120–140°C, organic acid was expelled from thermally mature source rocks and flowed into the Xiaoheba Formation sandstone, dissolving albite (an acidic plagioclase) to form kaolinite (Reaction 2).

$$2NaAlSi_{3}O_{8}\left(albite\right)+2H^{+}+H_{2}O=Al_{2}Si_{2}O_{5}{\left(OH\right)}_{4}\left(kaolinite\right)+4SiO_{2}\left(silica\right)+2Na^{+}$$ (2)

Illitization of kaolinite occurred in a closed system (temperatures in excess of 120–140°C) during the deep burial stage (Reaction 3). This process requires the consumption of K^+^, and the K^+^ source was mainly provided by potassium feldspar dissolution at that time (Reaction 4) [52].

$$3Al_{2}Si_{2}O_{5}{\left(OH\right)}_{4}\left(kaolinite\right)+2K^{+}=2KAl_{3}Si_{3}O_{10}{\left(OH\right)}_{2}\left(illite\right)+2H^{+}+3H_{2}O$$ (3)

$$2KAlSi_{3}O_{8}\left(potassium\;feldspar\right)+2H^{+}+H_{2}O=Al_{2}Si_{2}O_{5}{\left(OH\right)}_{4}\left(kaolinite\right)+4SiO_{2}\left(silica\right)+2K^{2+}$$ (4)

Combining Reaction 3 with Reaction 4 yields the following:

$$KAlSi_{3}O_{8}\left(potassium\;feldspar\right)+Al_{2}Si_{2}O_{5}{\left(OH\right)}_{4}\left(kaolinite\right)=2SiO_{2}\left(silica\right)+KAl_{3}Si_{3}O_{10}{\left(OH\right)}_{2}\left(illite\right)+H_{2}O$$ (5)

**Fig 10:**
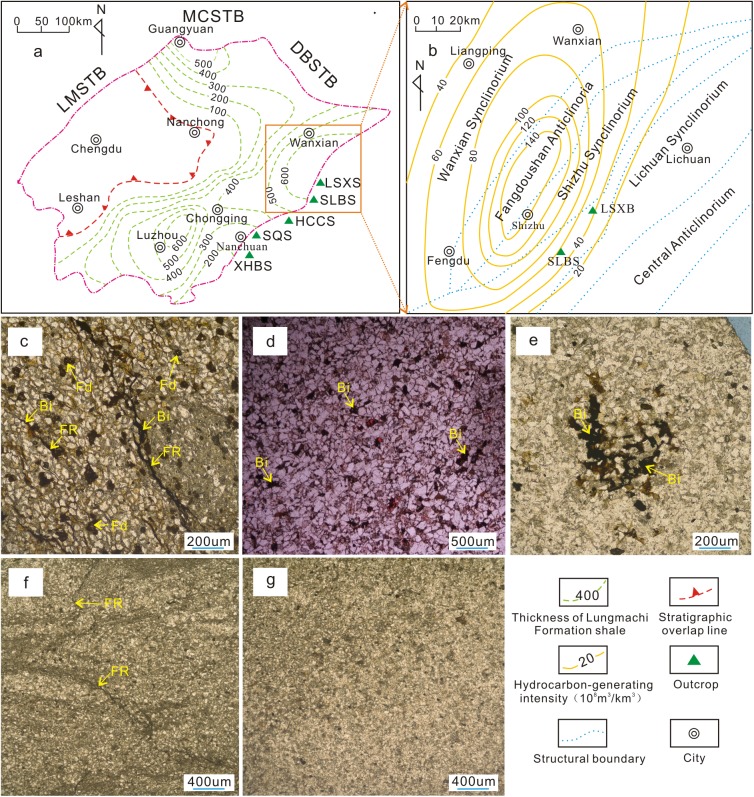
Composite figure showing the dissolution relationship between the Silurian Longmaxi source rock and the Xiaoheba Formation in the southeastern Sichuan Basin. a. Thickness of the Longmaxi shale in the Sichuan Basin (modified from Huang et al., 2012) [55]. b. Intensity of hydrocarbon generation in the Longmaxi Formation of the Shizhu region (modified from Wang, 2010) [56]. c. Secondary dissolution pores and fracture filled with bitumen, Shuangliuba section, Shizhu; d. Intergranular pores and moldic pores filled with bitumen, Lengshuixi section, Shizhu. e. Intergranular dissolved pores filled with bitumen, Huangcaochang section, Wulong. f. Common tight micro-fracture with no bitumen, Sanquan section, Nanchuan. g. Common tight sandstone with no bitumen filling, Xiaoheba section, Nanchuan. Fd-feldspar dissolution; Bi-bitumen; FR-fracture.

This phase does not consume H^+^, and Reaction 5 will occur continuously and automatically as long as kaolinite and potassium feldspar are available. The Xiaoheba Formation does not contain kaolinite but is rich in illite and pores of resulting from the dissolution of potassium feldspar and albite, indicating kaolinite produced by the early albite dissolution was completely transformed into illite during the deep burial period. During the deep burial stage, kaolinite was converted entirely into illite, and potassium feldspars grains were partially dissolved. Therefore, the potassium feldspar dissolution intensity during the later stage was directly dependent upon the organic acid dissolution intensity during the early stage. This conclusion is also supported by other robust evidence: sections close to the hydrocarbon generation center, such as the Shuangliuba, Lengshuixi, and Huangcaochang sections (Fig 10A and 10B), contain more secondary pores and common bitumen residues (Fig 10C, 10D and 10E). In contrast, the Sanquan and Xiaoheba sections, far from the hydrocarbon generation center, containing relatively fewer secondary pores and nearly no organic matter residue (Fig 10F and 10G). In addition, for a given sandstone feldspar content, the porosity of the Shuangliuba section (which is near the hydrocarbon generation center) is obviously higher than that of the Lengshuixi section (Fig 9B).

Hence, the formation of secondary porosity in the Xiaoheba sandstone reservoir is related to the dissolution of plagioclase by organic acids and the dissolution of potassium feldspar at high temperatures. The rise in pressure induced by hydrocarbon generation enabled a vertical migration of fluids and a resulting release of pressure [54], during the hydrocarbon generation and migration period of the underlying Longmaxi source rocks in the Late Permian to Late Triassic, This process produced acidic fluids that flowed through vertical fractures into the Xiaoheba sandstone, driving the dissolution of plagioclase in the sandstone. In addition, oil and gas charging preserved the dissolved pores. In the later period of deep burial, the illitization of kaolinite produced by the plagioclase dissolution consumed K^+^, resulting in the dissolution of potassium feldspar.

### 5.3. Connectivity of the fractures

The fractures in the Xiaoheba sandstone developed due to multi-stage tectonic activity during the Caledonian, Hercynian, Indosinian-Yanshanian, and Himalayan periods [57]. Based on the fracture and hydrocarbon charging relationships observed in the thin section at least three stages of fractures have been identified. The early-stage fractures are entirely filled with bitumen (Fig 5M), the medium-stage fractures are filled with calcite and some bitumen (Fig 5N), and the late-stage fractures remain unfilled (Fig 5O). The early-stage fractures are important as they served as favorable channels connecting the underlying source rock and the Xiaoheba Formation following hydrocarbon generation, Which enhanced the feldspar dissolution in the sandstones, thereby improving the quality of the reservoir. Dissolved pores are observed on both sides of the fractures and contain abundant bitumen (Fig 5M). The considerable amount of hydrocarbons generated by the underlying Longmaxi shale migrated into the Xiaoheba Formation, where they accumulated resulting in a rise in pressure. This pressure increase, combined with the fluid pressure caused by the poor drainage of the formation resulting from the seal formed by the overlying thick-bedded Hanjiadian mud shale, led to the development of hydraulic fractures, which also acted as a pathway for organic acid migration.

### 5.4. Formation mechanism and significance of high-quality reservoirs

The Xiaoheba sandstone is commonly tight, and the formation of the relatively high-quality reservoirs was related to three factors: microfacies, feldspar dissolution, and fractures. The delta front distal bar microfacies is interbedded with the interdistributary bay with the approximately vertical equal thicknesses and is relatively weakly compacted and cemented. Areas in close proximity to a hydrocarbon generation center, where the pressure induced by hydrocarbon generation enabled the vertical migration of organic acid fluids, are considered favorable for dissolution. Fracturing is an important factor in the formation of reservoirs. Fractures connecting the underlying source rock during the hydrocarbon generation and expulsion periods are particularly influential.

**Fig 11:**
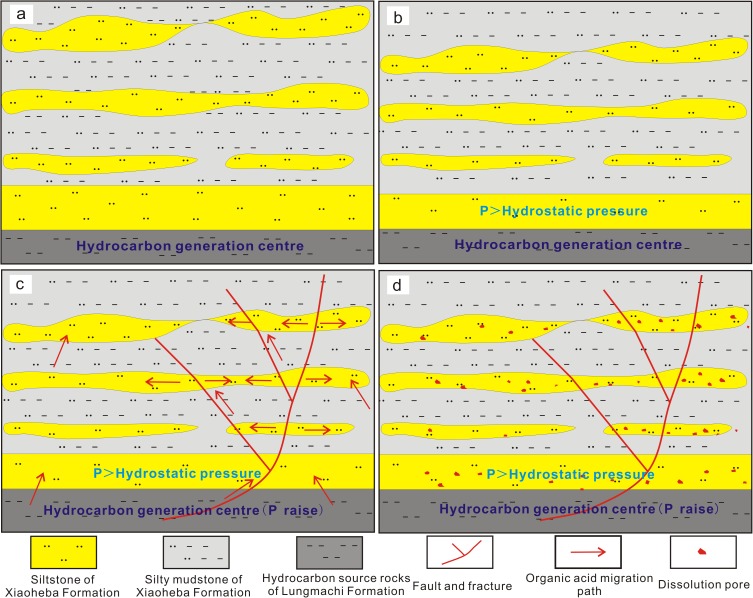
Formation model of a relatively high-quality reservoir in the Silurian Xiaoheba Formation in the southeastern Sichuan Basin.

Distal bar and delta front interdistributary bay deposits are represented by vertically superimposed sandstones and mudstones (Fig 11A), which were rapidly compacted after burial (Fig 11B). Silty mudstone and mud shale can be readily compacted due to the lack of grain support, resulting in a rapid decrease in permeability. However, the siltstone retains some primary pores because of the support from sandstone matrix grains. As the burial depth of the sandstone increases, the presence of silty mudstone and mudstone hinders the drainage of water from the sand bodies. At this point, the fluids inside the sand bodies bear some of the formation pressure, hindering further compaction. During the late Permian to Late Triassic, the Longmaxi organic matter became thermally mature and generated considerable organic acids and hydrocarbons. In the presence of large faults or fractures, organic acids continuously flowed into sand bodies and accumulated in the vicinity of structural fractures (Fig 11C). This process can cause the feldspar dissolution in sandstones, driving the formation of dissolution pores (Fig 11D).

The Xiaoheba Formation sediments were sourced from a delta depositional system situated in the southwestern segment of the Xufengshan uplift to the southeast [23, 24]. The delta front subfacies likely extends into the Zhongxian region, west of the Qiyueshan-Xishui fault. The Xiaoheba sandstone is moderately buried, contains good sandy microfacies (e.g., distal bar), and features a moderate sand-shale ratio in the vertical section, which led to relatively weak early-stage compaction and cementation. The close proximity of the Xiaoheba sandstone to the hydrocarbon generation center in the Longmaxi shale meant that organic acids continuously flowed into the sandstone generated Secondary pores, The weak deformation via folding and thrusting and the good sealing capability of the cap rock enables led to good preservation conditions [58], In the future, the area west of the Shuangliuba and Lengshuixi regions is the potential for gas exploration.

## 6. Conclusion

The Xiaoheba sandstone is classified as an ultra-tight and ultra-low permeability tight sandstone reservoir. This reservoir is present mainly in the Lengshuixi and Shuangliuba regions. The relatively high-quality portions of the reservoir are present in the Shuangliuba region. The reservoir space includes secondary intergranular dissolved pores, intragranular pores, moldic pores and fractures, with predominantly micro-scale pore throats. The pores are largely filled with bitumen, highlighting the potential of exploration. The reservoir quality is dependent on the microfacies, dissolution, and fracture connectivity. The delta front distal bar microfacies is interbedded with the interdistributary bay microfacies, and the deposits are of similar thickness. Relatively weak compaction and cementation has allowed for indirect preservation of primary intergranular pores, which enhanced later-stage dissolution. The late-stage potassium feldspar dissolution intensity was directly dependent on the early-stage organic acid dissolution. Areas closer to the hydrocarbon generation center experienced stronger dissolution activity. Early-stage fractures acts as migration pathways for organic acids and are considered an important condition for reservoir formation. The area to the west of the Shuangliuba and Lengshuixi regions has potential for gas exploration.
